# Coral-like Magnetic Metal–Organic Framework for Selective Adsorption and Detection of Thiabendazole in Tomato and Chinese Cabbage Samples

**DOI:** 10.3390/foods14213748

**Published:** 2025-10-31

**Authors:** Miao Wang, Xijuan Zhao, Zhihao Lin, Hailong Yu, Yanyan Huang, Bining Jiao, Jie Zhou, Ge Chen, Guangyang Liu, Lin Qin, Xinyan Liu, Donghui Xu

**Affiliations:** 1 Key Laboratory of Quality and Safety Control of Citrus Fruits, Ministry of Agriculture and Rural Affairs, Southwest University, Chongqing 400712, China; wmmwm001@163.com (M.W.); xijuanzh@swu.edu.cn (X.Z.); 2 State Key Laboratory of Vegetable Biobreeding, Institute of Vegetables and Flowers, Chinese Academy of Agricultural Sciences, Key Laboratory of Vegetables Quality and Safety Control, Ministry of Agriculture and Rural Affairs of China, Beijing 100081, China; zhihaol401@163.com (Z.L.); huangyanyan0412@163.com (Y.H.); zhoujiecaas@163.com (J.Z.); chenge@caas.cn (G.C.); qinlin01@caas.cn (L.Q.); liuxinyan@caas.cn (X.L.); xudonghui@caas.cn (D.X.); 3 National Center of Technology Innovation for Comprehensive Utilization of Saline-Alkali Land, Yingkou 257347, China; 4 School of Bioengineering, Beijing Polytechnic University, Beijing 100176, China; yuhailong_1978@163.com

**Keywords:** selective adsorption, thiabendazole, magnetic porous materials, ZIF-8, pesticide residues

## Abstract

The quality and safety of agricultural products are important factors in safeguarding human health and promoting sustainable agricultural development. However, for the purpose of improving the yield and quality, the misuse of pesticides often occurs, causing pesticide residues to remain in vegetables, posing threats to both the environment and human health. In order to detect and adsorb pesticide residues in vegetables, a coral-like novel magnetic porous nanomaterial (Fe@MDZ) was developed in this study as an adsorbent to adsorb thiabendazole (TBZ). Fe@MDZ has a large specific surface area (229.254 m^2^/g) and high saturation magnetization intensity (57.38 emu/g) with good adsorption capacity for TBZ. When the initial concentration was 2 mg/L, the adsorption capacity for TBZ was 1.23 mg/g. The static adsorption process matched the Langmuir isotherm model and was consistent with the pseudo-second-order kinetic model. In addition, the recovery of TBZ in both tomato and Chinese cabbage samples at different concentrations was above 70%. This work provides a new idea for the detection of TBZ pesticide residues in vegetables.

## 1. Introduction

The issue of food quality and safety is closely linked to our lives. The problem of pesticide residues in vegetables is a key issue in the quality and safety of agricultural products. Pesticides are an effective means of controlling pests and diseases in agricultural production. They have widely improved crop yields and quality [1]. However, the indiscriminate and unmanaged use of pesticides not only leads to the presence of agrochemical residues detected in vegetable crops, but also causes contamination of soil, sediment, and aquatic systems. These contaminants bioaccumulate and biomagnify along the food web, ultimately endangering the environment and affecting human health [2]. Thiabendazole (TBZ), a benzimidazole-class fungicide, functions both as a pre-harvest pesticide applied to vegetable crops and as a post-harvest protective agent that controls mold development, insect activity, decay, and maintains product quality throughout storage and shipping [3,4,5]. However, TBZ cannot be effectively extracted from fruits and vegetables by washing and remains stable during food processing, which makes it easy to form pesticide residues in vegetables, which are a threat to the health safety of the population and may lead to liver damage in severe cases [6,7]. Therefore, the detection and adsorption of pesticide residues is crucial.

The choice of pre-concentration technology is critical due to the complexity of the sample matrix and the wide variety of pesticides in fruits and vegetables, which can compromise the recovery rate and analytical accuracy [8,9]. Solid-phase extraction (SPE), a chromatographic sample pretreatment method, is recognized for its operational simplicity, low organic solvent consumption, and high enrichment, in which adsorbents are often used to separate, concentrate, and purify specific analytes [10]. Among the phenolic compounds, the second most abundant plant polyphenol is tannic acid (TA), environmentally friendly with an evanescent dendritic structure [11,12]. TA can interact with multiple compounds through covalent and non-covalent interactions, such as coordination with a variety of transition metals (e.g., iron, cobalt, or manganese) [13]. This means that TA is highly capable of adsorbing a wide range of substances. Magnetic nanoparticles, as environmentally friendly material, have unique magnetic force, and can be rapidly phased under an applied magnetic field, but unmodified magnetic nanoparticles are unstable, exhibit low surface energy, and have limited applications [14]. Therefore, metal-polyphenol networks (MPNs) with magnetic properties can be obtained by combining Fe_3_O_4_ nanoparticles with TA.

The functionalization of material surfaces is important for the development of science and technology [15]. Secreted by marine bivalves, dopamine hydrochloride (DA-HCl) acts as an adhesive protein. Its biocompatibility arises from the presence of diverse reactive moieties within its molecular structure [16]. Polydopamine (PDA) contains functional groups such as catechol and amino groups, enabling it to modify and functionalize a wide range of materials through non-covalent interactions [17,18]. Metal–organic frameworks (MOFs) can be functionalized to achieve desired host-guest interactions due to their excellent attributes and prospective utilizations in adsorption, separation, purification, and catalysis [19,20,21]. Compared with traditional adsorbents, MOFs are highly efficient adsorbents with diverse structural units, high porosity, flexible frameworks, and adjustable pore sizes [22,23]. Peng et al. developed a highly selective and sensitive luminescent sensor based on a Tb^3+^-functionalized Zr-MOF (Tb^3+^@1) for the detection of TBZ in oranges, which demonstrated a linear range of 0–80 μM, a detection limit of 0.271 μM, and a response time of less than 1 min [24]. Jia et al. developed an intelligent MOF-on-MOF hydrogel for detecting thiophanate-methyl in fruits and vegetables such as apples and cucumbers, which exhibited a broad linear range of 10 to 100 μM and a detection limit of 0.14 μM [25]. ZIF-8 is a category of MOFs with good chemical stability and flexibility in guest adsorption, making it an excellent candidate for pesticide adsorbents [26]. Reda M. Abdelhameed et al. found that the maximum adsorption capacity of ZIF-8 for prothiofos was 366.7 mg/g [27]. In addition, Meng et al. found that the maximum adsorption capacity of magnetic nanoparticles Fe_3_O_4_@ZIF-8@SiO_2_ for fenvalerate, β-cyfluthrin, and tetramethrin were 316.23, 364.43, and 258.69 mg/g, respectively [28].

**Figure 1:**
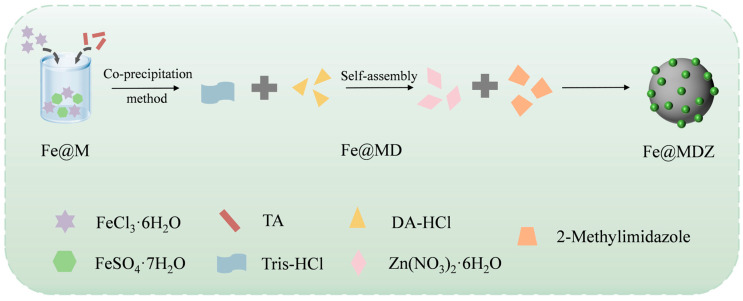
Preparation of Fe@MDZ.

In this work, a new coral-like magnetic porous nanomaterial (Fe@MDZ) was prepared by combining Fe_3_O_4_, TA, DA-HCl, and ZIF-8 with magnetic properties using the co-precipitation method as an adsorbent for removal of TBZ from tomato and Chinese cabbage (Figure 1). The impact of the initial adsorption concentration, adsorption time, adsorbent dose, and initial pH on TBZ removal efficiency as well as desorption solvent and time of adsorbent were investigated and optimized. The isotherm model and adsorption kinetics of Fe@MDZ were clarified, as well as the recoveries in tomato and Chinese cabbage. The correlation amidst the adsorption mechanism and performance between adsorbents and pesticides was discussed, providing new ideas for pesticide residue detection.

## 2. Materials and Methods

### 2.1. Materials

The sources used to synthesize Fe@MDZ were FeCl_3_·6H_2_O (96%) and FeSO_4_·7H_2_O (95%), which were purchased from Shanghai Macklin Biochemical Co., Ltd. (Shanghai, China). 2-Methylimidazole (C_4_H_6_N_2_, 99%) and Zn(NO_3_)_2_·6H_2_O (≥98%) were purchased from Shanghai Aladdin Biochemical Technology Co., Ltd. (Shanghai, China). Tannic acid (C_76_H_52_O_46_, 99%, Sinopharm Chemical Reagent Co., Ltd., Shanghai, China) and dopamine hydrochloride (C_8_H_12_ClNO_2_, ≥99.9%, J&K Scientific Co., Ltd., Beijing, China), all pesticides were procured from the Agro-Environmental Protection Institute, Ministry of Agriculture and Rural Affairs (Tianjin, China).

### 2.2. Instrument

The morphological structures of Fe@M and Fe@MD were observed by an SM-6300 scanning electron microscope (SEM), and the structure of Fe@MDZ was observed by a JEM-200CX transmission electron microscope (TEM) from JEOL, Tokyo, Japan. The functional groups of Fe_3_O_4_, ZIF-8, Fe@M, Fe@MD, and Fe@MDZ were detected and verified using a Nicolet iS10 Fourier Transform Infrared Spectrometer (FT-IR, Thermo Scientific, Waltham, MA, USA). The crystal structures of Fe_3_O_4_, ZIF-8, Fe@M, Fe@MD, and Fe@MDZ were verified using a D8 Advance X-ray diffractometer (XRD, Bruker AXS GmbH, Karlsruhe, Germany). The magnetic properties of the materials were evaluated using the Lake Shore 7401 Vibrating Sample Magnetometer (VSM, Lake Shore Cryotronics, Inc., Columbus, OH, USA). The MicroActive for ASAP 2460 equipment (Micromeritics Instrument Corp., Norcross, GA, USA) was used to analyze the specific surface area, pore volume, and pore size of Fe@MDZ using the Brunauer–Emmett–Teller (BET) method.

### 2.3. Preparation of Fe_3_O_4_@MPN

Initially, 0.60 g/L FeCl_3_·6H_2_O and 0.35 g/L FeSO_4_·7H_2_O were dissolved in 20 mL deionized water (DI water). The mixed solution was subsequently filtered through a 0.22 μm disposable needle-type filter and transferred into 240 mL of DI water. After magnetic stirring for 30 min (water bath at 80 °C), we added 10 mL of ammonia and continued stirring for 30 min. The material was cleaned with DI water and absolute ethanol (EtOH) by magnetic separation. It was then dispersed in 20 mL of DI water. After the addition of 10 g/L FeCl_3_·6H_2_O (0.4 mL), the mixture was stirred for 15 min, followed by the addition of 20 g/L TA (0.8 mL) and a further 30 min of stirring. Following magnetic separation, the solid material underwent sequential washing with DI water and EtOH. After vacuum drying to obtain Fe_3_O_4_@MPN (Fe@M).

### 2.4. Preparation of Fe_3_O_4_@MPN@PDA

The prepared Fe@M was added to a mixed solution containing 45 mL of Tris-HCl (10 mM, pH 8.5) and 5 mL of 20 g/L DA-HCl solution, followed by magnetic stirring for 24 h. Subsequently, the material was magnetically separated and washed with DI water and EtOH. The Fe_3_O_4_@MPN@PDA (Fe@MD) was obtained.

### 2.5. Preparation of Fe_3_O_4_@MPN@PDA@ZIF-8

The pre-synthesized Fe@MD nanocomposites were dispersed in 20 mL of DI water to form a homogeneous suspension. Separately, 0.30 g of Zn(NO_3_)_2_·6H_2_O was dissolved in 5 mL of DI water. Under continuous magnetic stirring, this solution was slowly added to the Fe@MD dispersion (stirred for 30 min). A solution of 0.82 g of 2-methylimidazole in 20 mL of DI water was then introduced into the reaction system, and mixing continued for 1 h. Washed with DI water and EtOH. Then dried in an oven at 60 °C for 12 h to obtain the Fe_3_O_4_@MPN@PDA@ZIF-8 (Fe@MDZ).

### 2.6. Adsorption Experiment

Following preparation of a 1000 mg/L TBZ stock solution in methanol, aqueous dilution was performed to achieve target working concentrations (0.1–15 mg/L). To carry out the adsorption experiments, 4 mL of TBZ solution of different concentrations was taken, and 5 mg of adsorbent was added, and the mixture was shaken for 30 min. The magnetic solid material in the solution was separated from the solvent using magnetic separation techniques, and then the supernatant was collected and filtered through a 0.22 μm disposable needle-type filter, and the concentration of TBZ in the filtered solution was detected with the help of HPLC-MS/MS (LCMS-8050, Shimadzu Corp., Kyoto, Japan).

The adsorption performance of Fe@MDZ was evaluated by adsorption capacity (Q) and adsorption rate (extraction efficiency). These values were derived using the following computational formula:

$$Q=\frac{\left(C_{0}-C_{e}\right)}{m}\times V$$

$$\mathrm{Extraction}\;\mathrm{efficency}=\frac{\left(C_{0}-C_{e}\right)}{C_{0}}\times 100\%$$

C_0_ (mg/L): the initial TBZ concentration;
C_e_ (mg/L): the solution concentration at the adsorption equilibrium state;
V (L): the volume of TBZ solution;
m (mg): the quantity of Fe@MDZ employed.

## 3. Results

### 3.1. Characterization

#### 3.1.1. TEM and SEM

**Figure 2:**
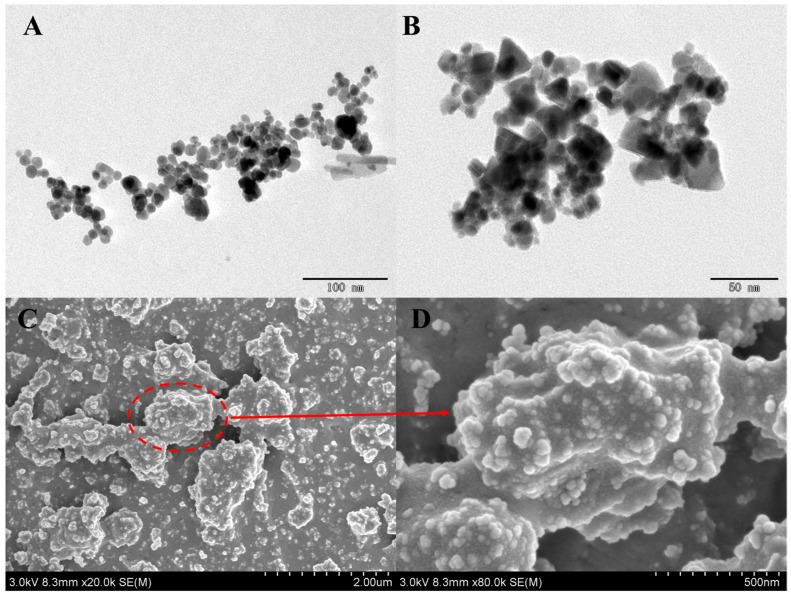
TEM images of Fe@M (**A**) and Fe@MD (**B**); SEM images of Fe@MDZ (**C**,**D**).

To be able to understand the morphology, structure, and particle size of Fe@MDZ composites in more detail, the materials were explored using SEM and TEM. Figure 2 graphically presents the experimental results, where the TEM image of Fe@M (Figure 2A) presents an irregular spherical shape, and after co-precipitation with DA-HCl (Figure 2B), the PDA coating with adhesive properties was successfully combined with the magnetic metal-polyphenol Fe@M material. Based on the SEM image of Fe@MDZ in Figure 2C, the data demonstrate that after Fe@MD was combined with ZIF-8, the material exhibited a coral-shaped irregular structure and porous conformation, indicating that the metal-polyphenol complexes were successfully combined with ZIF-8 with a porous structure through the co-precipitation synthesis method. Although the distribution of ZIF-8 on the surface of the material is not uniform, this alternating staggered inhomogeneous structure happens to increase the pore space of the material, thus improving the adsorption capacity of the composites for TBZ pesticide.

#### 3.1.2. XRD

**Figure 3:**
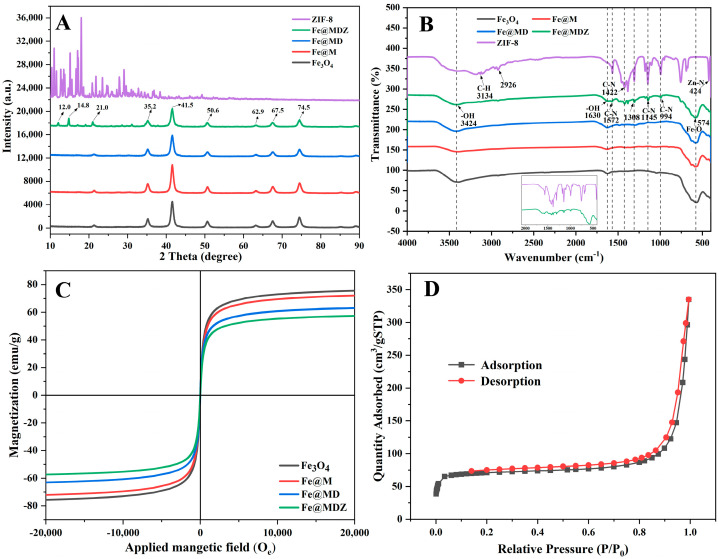
XRD (**A**); FT-IR (**B**); VSM (**C**); BET (**D**).

We conducted XRD characterization of Fe_3_O_4_, Fe@M, Fe@MD, and Fe@MDZ composites to probe their phase composition and structural integrity. It can be observed in Figure 3A, in which five characteristic peaks of Fe_3_O_4_ nanoparticles appeared at 35.2°, 41.5°, 50.6°, 62.9°, and 74.5°, which correspond to the reflectance patterns of (311), (400), (422), (440), and (002) in the standard card JCPDS No. 65-3107, respectively [29,30]. According to the results of XRD characterization of other materials, the XRD pattern exhibits distinct characteristic peaks corresponding to Fe_3_O_4_ nanoparticles after modification of MPN and PDA still clearly appear in similar positions, indicating that the self-assembly of MPN and the self-polymerization of PDA did not destroy the structure of Fe_3_O_4_. The Fe@MDZ composites showed prominent reflection peaks at 12.0° and 14.8°, and the crystal planes were (002) and (112), which were the characteristic peaks of ZIF-8 material by comparing them with the JCPDS standard card and the characterization results of ZIF-8 in Figure 3A, indicating that ZIF-8 nanoparticles were successfully combined with Fe@MD material [31]. While the peak strength of the composites is decreasing with the layer-by-layer modification of MPN and PDA and the growth of ZIF-8 crystals on Fe@MD materials, which may be attributed to the gradual decrease in the amount of Fe_3_O_4_ [32].

#### 3.1.3. FTIR

Figure 3B illustrates that the FTIR characterization results of the five materials prepared in the experiment have a bonding range between 4000 and 400 cm^−1^. The infrared spectra of Fe_3_O_4_ nanoparticles showed that the broad peak at 3424 cm^−1^ may be the stretching vibration of -OH groups on the surface of Fe_3_O_4_ nanoparticles, 1630 cm^−1^ was caused by the bending vibration of -OH, and the peak at 574 cm^−1^ belongs to the Fe-O stretching vibration of Fe_3_O_4_, which was in agreement with previously reported results [33,34]. Similar characteristic peaks appeared in the IR spectrograms of Fe@M, Fe@MD, and Fe@MDZ materials, but the intensities of these characteristic peaks were gradually weakening, which may be due to the capping of MPN and PDA, which made the structure of Fe_3_O_4_ nanoparticles exist but become less in number, which is in agreement with the characterization in XRD. This is consistent with the results in XRD, which proves the successful coating of the composites [35]. Based on the spectral image of ZIF-8, the experimental data confirm that the characteristic peak at 3134 cm^−1^ belongs to the stretching vibrational mode of the aromatic C-H bond, and the stretching mode of the C-H bond of the imidazolyl moiety and the methyl substituent in the linker is observed at 2926 cm^−1^ [36]. The peaks located at 1572 cm^−1^, 1422 cm^−1^, 1145 cm^−1^, and 994 cm^−1^ correspond to the stretching vibrations of the C-N bond. The characteristic peak located at 424 cm^−1^ is the stretching vibrational mode of the Zn-N bond. The other peaks below 1308 cm^−1^ are in-plane and out-of-plane bending modes of the imidazole ring [37]. Therefore, the four newly appeared peaks at 1572 cm^−1^, 1308 cm^−1^, 1145 cm^−1^, and 994 cm^−1^ in the Fe@MDZ composite are the characteristic peaks of ZIF-8, which verifies that the ZIF-8 crystals were effectively grown on top of the composite.

#### 3.1.4. VSM

Different shapes, sizes, and crystal structures have a profound effect on saturation magnetization strength of nanostructures of magnetic materials [30]. Therefore, experimentally, hysteresis regression lines were measured by VSM to check the magnetic properties of the prepared composites, Figure 3C shows the VSM hysteresis regression lines of different materials. The results show that the hysteresis loops of Fe_3_O_4_, Fe@M, Fe@MD, and Fe@MDZ are free of hysteresis, remanent magnetism, and have a coercivity close to zero, which exhibits typical superparamagnetic behavior, where the maximum saturation magnetization strength of Fe_3_O_4_ nanoparticles is 75.64 emu/g, which decreases to 72.14 emu/g in Fe@M and continues to decrease to 63.25 emu/g in Fe@MD, and finally 57.38 emu/g in the Fe@MDZ composite. Thus, on account of the magnetic characteristics of Fe@MDZ, the nanoparticles can be quickly divided from the solvent with the aid of an external magnetic field. When the applied magnetic field disappears, the nanoparticles are immediately dispersed in the solvent with a slight shake, which facilitates the collection, regeneration, and reuse of the material [38,39].

#### 3.1.5. BET

The isotherms are highly sensitive to pore size, structural inhomogeneity, and adsorbent-adsorbent interactions [40]. From Figure 3D, the N_2_ adsorption–desorption isotherms of Fe@MDZ composites were accompanied by hysteresis loops after capillary coalescence, and hysteresis loops with relative pressures ranging from 0.20 to 0.95 were caused by wedge capillary structures, which were related to the occurrence of capillary shrinkage in the pores [41]. The specific surface areas associated with ZIF-8, Fe@MD, and Fe@MDZ were 276.996, 93.807, and 229.254 m^2^/g; the pore volumes were 0.168, 0.233, and 0.238 cm^3^/g; and the pore sizes were 2.428, 9.947, and 4.154 nm, respectively (Table 1). These data indicate that the Fe@MDZ composite has a mesoporous structure and high specific surface area, which helps to increase the adsorption capacity of the composites [42].

#### 3.1.6. XPS and ICP-OES

**Figure 4:**
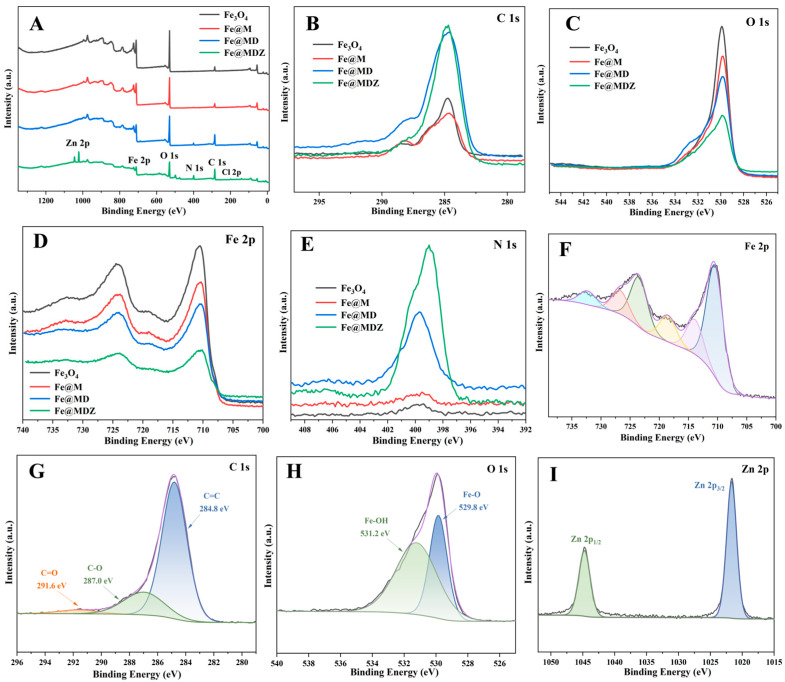
(**A**) Full-scan XPS spectra of Fe@MDZ-related samples; High-resolution XPS spectra of Fe@MDZ-related samples: (**B**) C 1s, (**C**) O 1s, (**D**) Fe 2p, (**E**) N 1s; (**F**–**I**) XPS spectra of Fe 2p, C 1s, O 1s, and Zn 2p peaks for Fe@MDZ.

Figure 4A displays the full XPS scans of Fe@MDZ and related materials. The results indicate the presence of C, N, O, Cl, and Fe in Fe_3_O_4_, Fe@M, and Fe@MD, while Fe@MDZ, which incorporated ZIF-8, also contains Zn. The results revealed (Figure 4B–E) that following PDA modification, the peak intensities of C 1s and N 1s increased significantly, whereas those of O 1s and Fe 2p diminished. This may be attributed to the self-polymerization of catecholamines within the PDA layer on the Fe@M surface, which attenuates the signals from O 1s and Fe 2p [43]. Following the introduction of ZIF-8, a new Zn 2p peak emerged and the intensity of the N 1s peak increased significantly, while the intensities of the O 1s and Fe 2p peaks decreased. This may relate to the Zn-O bonds and imidazole rings present in ZIF-8 [44,45]. In the XPS spectra of Fe@MDZ for Fe 2p, C 1s, O 1s, and Zn 2p (Figure 4F–I), the Fe 2p_3/2_ peak was deconvoluted into two components at binding energies of 710.5 eV and 713.9 eV, while the Fe 2p_1/2_ peak was fitted with components at 723.4 eV and 726.7 eV. A satellite peak was also identified at 718.5 eV [46]. In the C 1s spectrum, binding energies of 284.8 eV, 287.0 eV, and 291.6 eV were observed, which may be attributed to C-O and C=O bonds based on literature reports for PDA and ZIF-8 [43,47]. The O 1s spectrum shows peaks at 529.8 eV and 531.2 eV, corresponding to Fe-O and Fe-OH bonds, respectively. Furthermore, ICP-OES results (Table 2) revealed a gradual decrease in the mass fraction of Fe with the successive incorporation of PDA and ZIF-8. Concurrently, Zn was detected, which is consistent with the XPS findings. Additionally, the results from elemental analysis (Table S2) are in accordance with this interpretation. These results collectively confirm the successful preparation of Fe@MDZ.

### 3.2. Adsorption Properties of Fe@MDZ

#### 3.2.1. Identification of Pesticides and Selection of Materials

**Figure 5:**
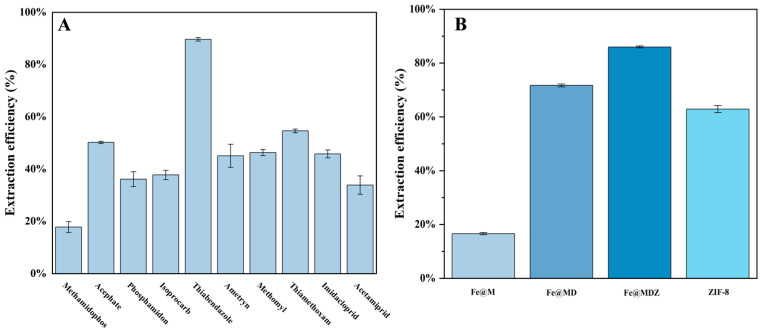
Adsorption of different pesticides by Fe@MDZ (**A**); adsorption of TBZ by different materials (**B**).

Experiments were carried out to adsorb five mg of Fe@MDZ material on ten pesticides, namely TBZ, Methamidophos, Acephate, Phosphamidon, Isoprocarb, Ametryn, Methomyl, Thiamethoxam, Imidacloprid, and Acetamiprid (at a concentration of 0.1 mg/L). These ten pesticides were adsorbed, and the results are summarized in Figure 5A. It was shown by the results that Fe@MDZ had the best adsorption efficiency for TBZ. To achieve better adsorption of TBZ by the adsorbent, the adsorption properties of 5 mg of Fe@M, Fe@MD, Fe@MDZ, and ZIF-8 materials on TBZ (concentration of 0.5 mg/L) were experimentally investigated. Data from Figure 5B showed that the extraction efficiency of Fe@M for TBZ was only 16.59%, while it significantly increased to 71.68% after the addition of PDA. After the addition of ZIF-8, the extraction efficiency of Fe@MDZ for TBZ reached 85.97%, which is also higher than that of ZIF-8 alone (62.88%). This may be attributed to the addition of ZIF-8, which increased the specific surface area of Fe@MDZ (Table 1), significantly expanding the number of adsorption sites on its surface and thereby enhancing the extraction efficiency of Fe@MDZ. Therefore, Fe@MDZ was ultimately selected as the adsorbent for TBZ.

#### 3.2.2. Effect of Initial Concentration and Adsorption Isotherm Analysis

**Figure 6:**
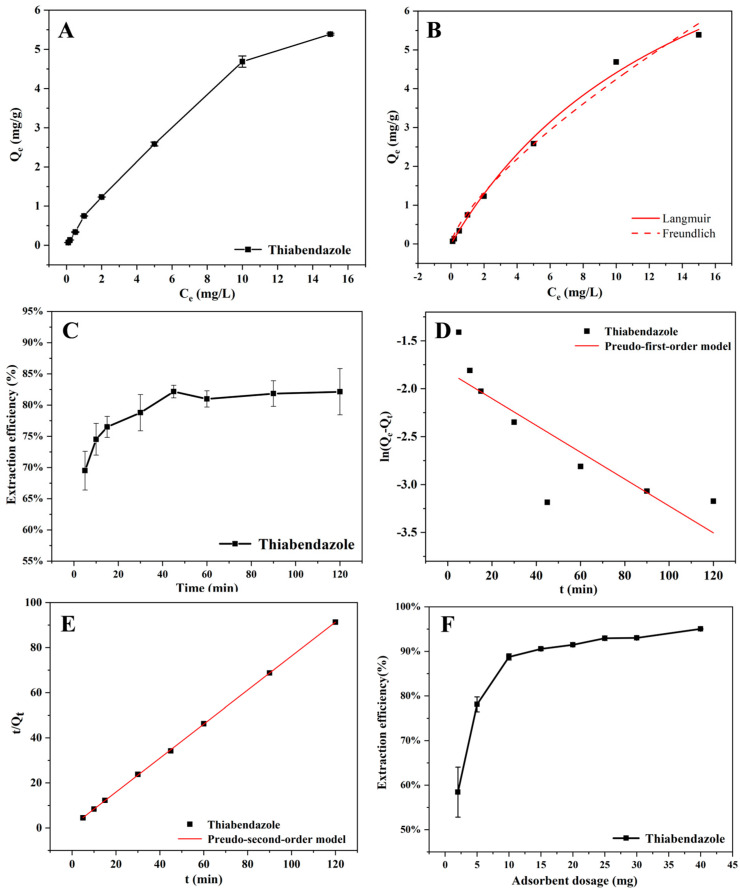
Adsorption capacity of TBZ on Fe@MDZ (**A**); Adsorption isotherms fitted to TBZ by Fe@MDZ (**B**); effect of time on the extraction efficiency of TBZ (**C**); adsorption kinetics of TBZ by Fe@MDZ fitted curve (PFO (**D**), PSO (**E**)); influence of Fe@MDZ dosage on TBZ extraction efficiency (**F**).

The impacts of various initial concentrations on the adsorption procedure were investigated, and 0.1, 0.2, 0.5, 1, 2, 5, 10, and 15 mg/L TBZ solutions were configured, 0.005 g of Fe@MDZ was weighed, 4 mL of different concentrations of TBZ solutions were added, shaking at the temperature of the room environment for 0.5 h. After the process of magnetic separation, the supernatant was collected. The supernatant underwent filtration through a 0.22 μm disposable needle-type filter and was then diluted with DI water. Subsequently, it was determined by HPLC-MS/MS mass spectrometer (Figure 6A,B). Across the concentration range of 0.1–10 mg/L, the adsorbent demonstrated a rapid increase in TBZ adsorption capacity as the concentration rose, exhibiting favorable adsorption performance. The observed adsorption behavior might stem from the presence of hydrogen bonding and electrostatic interactions between pesticide molecules and oxygen-containing functional groups on the adsorbent surface. However, at 10–15 mg/L, the adsorption capacity increased slowly and tended to be saturated. Combining the material properties, adsorption effects, and experimental losses, the TBZ pesticide concentration of 2 mg/L has been chosen as the initial concentration for the experiment, which was used for the subsequent experiments to explore the optimal conditions.

Understanding the effect of different adsorption processes on adsorbent behavior can be aided by adsorption isotherm models [48]. To be able to investigate the equilibrium adsorption phenomenon of TBZ pesticides on Fe@MDZ composites, experiments were carried out using Freundlich and Langmuir isotherm models. The mechanisms through which substances accumulate at material interfaces are systematically described by adsorption isotherm models, where Langmuir isotherms help determine the maximum adsorption capacity achievable on the adsorbent surface at saturation, and Freundlich isotherms are useful in understanding the energy required to adsorb when interacting with different adsorbents [49]. The Langmuir model facilitates the prediction of both the adsorbent’s theoretical maximum adsorption capacity and the interactive dynamics between the target adsorbate and environmental parameters during the adsorption process. Refer to Formula (S1) in the Supplementary Materials.

R_L_ is the Langmuir separation factor, which can be derived from the model. Adsorption process viability can be systematically determined through analysis of this dimensionless value. The R_L_ value falling within the range of 0 to 1 implies that the adsorption process is progressing favorably. When R_L_ = 0, it signifies an irreversible adsorption course; if R_L_ = 1, it shows a linear connection during the adsorption; and in the case of R_L_ > 1, it demonstrates that the adsorption process is heading in an unfavorable way. The formulae for the calculation of R_L_ with respect to K_L_ and C_0_ are shown in Formula (S2) in the Supplementary Material.

The Freundlich adsorption isotherm, which varies according to the system material and temperature, is employed to predict the adsorption amount at different concentrations. This model elucidates molecular-level mechanisms governing sorbent-environment phase interactions, thereby facilitating the rational design of porous materials with enhanced contaminant adsorption capacity. In this model, adsorption occurs on the surface of the adsorbent, which is inherently non-uniform and non-homogeneous in phase, thus forming a multilayer coverage on the surface [50,51]. The formula can be found in Formula (S3) in the Supplementary Material.

The interaction between TBZ pesticide and Fe@MDZ adsorbent was explored in relation to the adsorption parameters based on Langmuir and Freundlich adsorption isotherm models. The data obtained from experiments are confirmed in Figure 6B and Table 3. The results indicated that by comparing the R^2^ correlation coefficients of the two models, the R^2^ value of the Langmuir isotherm model is 0.99, which makes the model better suited to delineating the TBZ uptake mechanism by Fe@MDZ, indicating that the adsorption mainly occurs in a single-layer form. Moreover, the 0 < R_L_ < 1 indicates that TBZ pesticides on Fe@MDZ composites are favorable for the adsorption process. These results are useful in helping us to better predict and analyze the adsorption behavior of adsorbents for TBZ pesticides.

#### 3.2.3. Effect of Time and Analysis of Adsorption Kinetics

The influence of the contact time ranging from 5 to 120 min on the adsorption of TBZ pesticides by the Fe@MDZ composite material was studied (Figure 6C). The analytical results demonstrate that in 5–15 min, the adsorption rate increases rapidly and reaches 76.5%; in 15–45 min, the rate of adsorption decreases gradually, and the adsorption rate is 82.16% in 45 min. However, beyond 45 min, the adsorption efficiency of the composite material on TBZ pesticide is basically unchanged. The adsorption efficiency of the composites on TBZ pesticide was basically unchanged after 45 min, and the adsorption was in a stable stage. Therefore, 45 min was chosen as the optimum adsorption time.

For the determination of the adsorption kinetics of TBZ on Fe@MDZ composites under the present experimental conditions, we performed the fitting of pseudo-first-order (PFO) and pseudo-second-order (PSO) kinetic models. These two models are empirical models, originating from the kinetics of chemical reactions in homogeneous systems, and are applicable to non-homogeneous systems [52]. The kinetic equation assumes it is premised on the assumption that adsorption is a dual-stage process in which molecules first attach to a surface by adsorption, subsequently undergoing chemical reactions to form stronger bonds. The model can be used to better understand and analyze the adsorption process by measuring the adsorption rate and predicting the number of molecules likely to be adsorbed over time [53].

The PFO equation posits that the fraction of occupied adsorption sites is directly related to the quantity of available, unoccupied sites [54]. PSO models are capable of describing models of species adsorption in solution, measuring the extent of adsorption. Conceptualizing the model in this way indicates that in the adsorption process, which can be demonstrated by monolayer or multilayer adsorption or irreversible adsorption/desorption cycles. Specific formulas are given in the Formulas (S4) and (S5) in the Supplementary Material.

From the findings of the experiments (Figure 6D,E and Table 4), the PSO kinetic model correlation coefficient (R^2^) of 0.99 for TBZ is more consistent with the PSO model over the PFO model with R^2^ = 0.66, with chemisorption occurring predominantly. The calculated equilibrium adsorption capacity shows a close match with the experimental data, suggesting that the PSO kinetic model is better suited for characterizing the adsorption process of TBZ onto the Fe@MDZ composite material.

#### 3.2.4. Impact of Adsorbent Dose

The adsorption effect of Fe@MPZ on TBZ was investigated at the dose of 0.002–0.040 g (Figure 6F). At 0.002–0.010 g, the adsorption rate increased rapidly to 88.75%, and at 0.015–0.040 g, the adsorption efficiency gradually became flat. During the initial stage, the adsorbent’s large specific surface area and increased dosage lead to an abundance of accessible adsorption sites, thereby significantly enhancing adsorption efficiency. However, when the Fe@MPZ dose was further increased, the adsorption efficiency showed no significant change. This can be explained by the constant initial TBZ concentration, as adsorption proceeded, the TBZ concentration gradually decreased and eventually stabilized, limiting further improvements in efficiency despite the higher adsorbent dosage. Therefore, an adsorbent dose of 0.015 g was determined as the optimal, and the adsorption efficiency was 90.55%.

#### 3.2.5. Influence of pH on Adsorption

**Figure 7:**
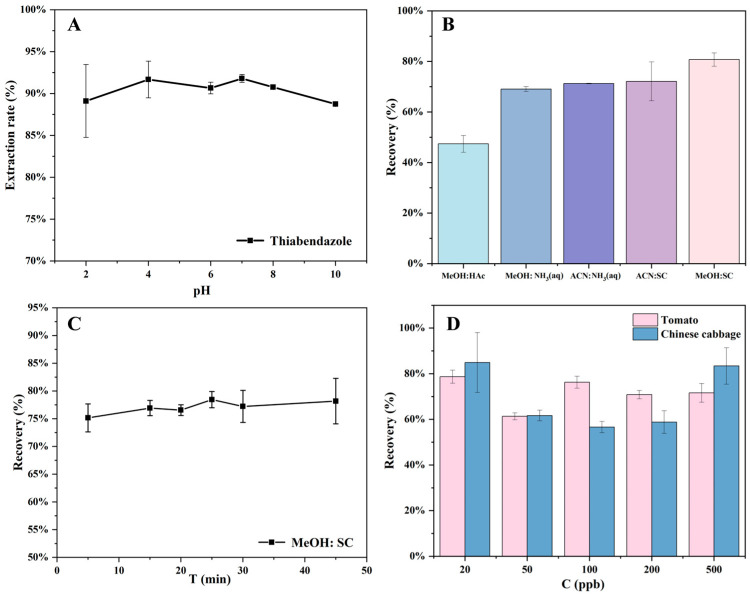
pH on the extraction efficiency for TBZ (**A**); effect of desorption solvent and time (**B**,**C**); real samples (**D**).

The solution pH regulates both the adsorbent surface charge and the adsorbate ionization degree, thereby influencing the maximum removal efficiency [55]. In this experiment, the adsorption of TBZ on composites in the pH range of 2–10 was investigated while keeping other experimental parameters unchanged (Figure 7A). At pH 2 and 10, the adsorption efficiency fluctuated slightly, to 88.75 and 89.10%, respectively. Under other pH conditions, it was above 90.00%, indicating that it had little effect on the adsorption effect. For follow-up experiments, pH = 7 was therefore chosen as the optimum condition.

#### 3.2.6. Effect of Desorption Solvents and Optimization of Time

In order to develop green and environmentally friendly adsorption materials and reduce the treatment cost, in-depth experimental investigations on the desorption of the adsorbent have been carried out. Experiments were designed to investigate the effect of different desorption solvents and the time of use of desorption solvents on the adsorption efficiency. Methanol:Acetic acid (MeOH:Hac, 9:1, V/V), Methanol:Ammonia water (MeOH:NH_3_(aq), 8:2, V/V), Acetonitrile:Ammonia water (ACN:NH_3_(aq), 8:2, V/V), Acetonitrile:Sodium citrate (ACN:SC, 7:3, V/V), and Methanol:Sodium citrate (MeOH:SC, 6:4, V/V) were selected as desorption solvents to analyze the adsorbed composites, and the results are demonstrated in Figure 7B. From left to right, the recovery rates were 47.36%, 69.05%, 71.25%, 72.09%, and 80.74%, respectively, and it can be seen that MeOH:SC (6:4, V/V) had the highest recovery of TBZ when it was employed as the desorption solvent. Therefore, MeOH:SC (6:4, V/V) was identified as the optimal choice. Furthermore, desorption time was also investigated (Figure 7C), and it was found that desorption time had little effect on Fe@MPZ recovery, and 20 min was validated as the optimum desorption time for comprehensive consideration.

### 3.3. Real Samples

To evaluate the adsorption characteristics of Fe@MDZ nanocomposites in authentic agricultural matrices including tomato and Chinese cabbage, TBZ contaminated samples with concentration gradients were subjected to experimental analysis. Fresh tomato and Chinese cabbage samples were chopped and processed using a blender. Subsequently, 10× *g* of each homogenized sample was weighed, centrifuged, and the supernatant was collected and diluted with 10 mL of DI water. Target solutions were prepared by spiking with TBZ at varying levels (0.02–0.5 mg/L). Fe@MDZ was weighed and dispersed into 4 mL of the prepared TBZ solution, and the reaction was conducted under the optimized conditions. Consequently (Figure 7D), when TBZ at 0.02–0.5 mg/L was added to tomato and Chinese cabbage samples, the recoveries of tomato samples ranged from 70.70 to 81.29%, with the highest recovery rate of 81.29% at 0.1 mg/L. For the Chinese cabbage samples, the recoveries ranged from 73.00 to 86.60%, with the highest recoveries at 0.02 mg/L, all within the acceptable range. In addition, comparisons were made with methods for detecting TBZ residues in other foods (Table 5). Vegetable substrates are relatively complex. In this study, the synthesis method of Fe@MDZ was simple, and the magnetic solid-phase extraction technology used was quick to operate and had good detection limits. In summary, Fe@MDZ has certain application value as an adsorbent for the extraction of TBZ from tomato and Chinese cabbage.

## 4. Conclusions

In this work, we prepared a porous magnetic material, Fe@MDZ, with a coral-like structure by the co-precipitation method, and obtained an adsorbent with a large specific surface area (229.254 m^2^/g), good pore volume (0.238 cm^3^/g), and superparamagnetism by coating a PDA layer on the surface of the Fe@MPN and combining it with ZIF-8. When Fe@MDZ adsorbs pesticide TBZ as an adsorbent, it is almost unaffected by pH and ion concentration. In addition, the adsorption isothermal model and adsorption kinetics demonstrated that the adsorption process of TBZ pesticides was more in line with the Fe@MDZ, the Langmuir isotherm model, and the PSO kinetic model, and the main occurrence was the monolayer chemisorption process. This may be related to π-π interactions and hydrogen bonding between the adsorbent and the material. Hence, this study established a pretreatment extraction and detection method for TBZ pesticide in tomatoes and Chinese cabbages. The method has good adsorption performance and specific recognition ability, offering a novel approach for the detection of TBZ residues in tomato and Chinese cabbage samples.

## Figures and Tables

**Table 1:** Gas adsorption data for ZIF-8, Fe@MD and Fe@MDZ.

Material	Surface Area (m^2^/g)	Pore Volume (cm^3^/g)	Pore Size (nm)
ZIF-8	276.996	0.168	2.428
Fe@MD	93.807	0.233	9.947
Fe@MDZ	229.254	0.238	4.154

**Table 2:** ICP-OES analysis results for Fe@MDZ-related materials.

Materials	Element	Content (mg/kg)	Wt.%
Fe_3_O_4_	Fe	657,419.0	65.74
Fe@M	Fe	647,209.6	64.72
Fe@MD	Fe	588,628.8	58.86
Fe@MDZ	Fe	451,265.8	45.13
	Zn	65,886.1	6.59

**Table 3:** Comparison of adsorption isotherm parameters.

Pesticide	Langmuir Equation				Freundlich Equation		
	Q_m_ (mg/g)	K_L_ (L/mg)	R^2^	R_L_	K_F_ (L/mg)	*n*	R^2^
TBZ	1.23	0.07	0.995	0.88	0.80	1.39	0.987

**Table 4:** Kinetic fitting parameters.

	Pseudo-First-Order Model				Pseudo-Second-Order Model		
	K_1_ (1/min)	Q_e,exp_ (mg/g)	Q_e,cal_ (mg/g)	R^2^	K_2_ (1/min)	Q_e,cal_ (mg/g)	R^2^
TBZ	0.014	1.36	0.16	0.66	0.673	1.33	0.99

**Table 5:** Compared with other methods for determining TBZ.

Sample	Method	Detection Technology	LOD	R^2^	Reference
Apple	Solid phase extraction	HPLC-FLD	7.5 ng/g	0.9998	[56]
Edible oil	Magnetic solid phase extraction	UHPLC-MS/MS	0.001 mg/kg	0.9957	[57]
Apple peel	Surface-Enhanced Raman Spectroscopy	/	0.20 g/cm^2^	/	[58]
Pear peel			40 ng/cm^2^		
Fruits and vegetables	Ionic liquid phase microextraction	Spectrophotometry	0.1, 0.24 μg/L	0.998–0.975	[59]
Wastewater	/	HPLC	0.0045 mg/L	0.999	[60]
*Ophiopogonis japonicus*	Pipette-tip solid-phase extraction	HPLC-UV	0.004 µg/g	0.999	[61]
Tomato and Chinese cabbage	Magnetic solid phase extraction	HPLC-MS/MS	0.5 μg/L	0.9914	This work

## Data Availability

The original contributions presented in this study are included in the article/Supplementary Materials. Further inquiries can be directed to the corresponding authors.

## Supplementary Materials

The following supporting information can be downloaded at: https://www.mdpi.com/article/10.3390/foods14213748/s1, Table S1: The linear equation, linear range, correlation coefficient and LOD of the TBZ detection method; Table S2: Elemental analysis of Fe@MDZ-related materials; References [62,63,64,65] are cited in the supplementary materials.
