# Supplementary material for: Coral-like Magnetic Metal–Organic Framework for Selective Adsorption and Detection of Thiabendazole in Tomato and Chinese Cabbage Samples
Source: Foods. 2025 Oct 31;14(21):3748. doi: 10.3390/foods14213748 (PMC12607549; doi:10.3390/foods14213748)
Supplement: Supplementary file 1 [file foods-14-03748-s001.zip › foods-3897436-supplementary.pdf]

Supplementary Materials

Coral-like Magnetic Metal-Organic Framework for Selective Adsorption and Detection of Thiabendazole in Tomato and Chinese Cabbage Samples

Miao Wang [1, 2, 3, †], Xijuan Zhao [1, †], Zhihao Lin [2], Hailong Yu [4], Yanyan Huang [2], Bining Jiao [1, *], Jie Zhou [2], Ge Chen [2], Guangyang Liu [2, 3, *], Lin Qin [2], Xinyan Liu [2], Donghui Xu [2, 3, *]

S1. Formulas

Formula (S1) [1]:

$$Q_e = \frac{Q_m K_L C_e}{1 + K_L C_e}$$

Q_e (mg/g): the adsorption equilibrium amount or adsorption capacity of the adsorbent;

Q_m (mg/g): the maximum adsorption capacity of the adsorbent;

K_L (L/mg): the Langmuir constant;

C_e (mg/L): the equilibrium adsorption concentration.

Formula (S2) [2]:

$$R_L = \frac{1}{1 + K_L C_0}$$

Formula (S3) [3]:

$$Q_e = K_F C_e^{\frac{1}{n}}$$

Q_e (mg/g) denotes the equilibrium adsorption capacity;

C_e (mg/L) represents the equilibrium concentration;

n and K_F (L/mg) are the adsorption equilibrium constants, which denote the adsorption capacity and adsorption strength. If $n<1$, the adsorption process follows chemisorption.

Formulas (S4) and (S5) [4]:

$$\ln(Q_e - Q_t) = \ln Q_e - K_1 t$$

$$\frac{t}{Q_t} = \frac{1}{K_2 Q_e^2} + (\frac{1}{Q_e})t$$

Q_t (mg/g): Amount of pesticide adsorbed at time t;

Q_e (mg/g): Amount of pesticide adsorbed at equilibrium;

K_1 [g/(mg·min)] and K_2 [g/(mg·min)] are the rate constant.

S2. Methodological validation

As shown in Table 1, when S/N=3, the limit of detection (LOD) of this method is 0.5 μg/L, and the standard curve R^2 is 0.9914. This indicates that the limit of detection in this study is low and the linear relationship is good.

Table S1 The linear equation, linear range, correlation coefficient and LOD of the TBZ detection method

Pesticide	Linear equation	Linear range (μg/L)	R^2	LOD (μg/L)
TBZ	Y=(15912.0)X-3192.38	1-1000	0.9914	0.5

S3. Elemental analysis

Table S2 Elemental analysis of Fe@MDZ-related materials

**Materials**	**N%**	**C%**	**H%**
Fe@M	0	1.55	0.530
Fe@MD	0.66	5.85	0.772
Fe@MDZ	5.30	12.46	1.475

References

[1] Li. J; Luo. T; Yan. W; Cheng. T; Cheng. K; Yu. L; Cao. J; Yang. Z. Adsorption Behavior and Adsorption Dynamics of Micrometer-Sized Polymer Microspheres on the Surface of Quartz Sand. *Processes*. **2023**, *11, 1432*. https://doi.org/10.3390/pr11051432.

[2] Radoor. S, Karayil. J, Parameswaranpillai. J, Siengchin. S. Removal of anionic dye Congo red from aqueous environment using polyvinyl alcohol/sodium alginate/ZSM-5 zeolite membrane. *Scientific Reports*. **2020**, *10, 15452*. https://doi.org/10.1038/s41598-020-72398-5.

[3] Ajith. A, Gowthaman. N.S.K, John. S.A, Elango. K.P. Direct Adsorption of Graphene Oxide on a Glassy Carbon Electrode: An Investigation of Its Adsorption and Electrochemical Activity. *Langmuir*. **2023**, *39, 9990-10000*. https://doi.org/10.1021/acs.langmuir.3c00768.

[4] Sodkouieh. S.M, Kalantari. M, Shamspur. T. Methylene blue adsorption by wheat straw-based adsorbents: Study of adsorption kinetics and isotherms. *Korean Journal of Chemical Engineering*. **2023**, *40, 873-881*. https://doi.org/10.1007/s11814-022-1230-0.
